# HIV/AIDS epidemic in Turkey and use of antiretroviral drugs for treating pregnant women and preventing HIV infection in infants

**DOI:** 10.4274/tjod.52128

**Published:** 2015-12-15

**Authors:** Çağrı Gülümser, Tuğrul Erbaydar

**Affiliations:** 1 Ankara University Faculty of Medicine, Department of Public Health, Ankara, Turkey

**Keywords:** AIDS, HIV, antiretroviral drugs, Pregnancy, infants, Turkey

## Abstract

**Objective:**

To evaluate changes in epidemiologic characteristics for HIV/AIDS in Turkey since 1985, management of HIV-positive pregnancies, and how new-borns and infants would be protected by anti-viral therapy (AVT).

**Materials and Methods:**

The World Health Organization (WHO) progress reports, 2013 UNAIDS Global AIDS epidemic reports, Turkish Ministry of Health HIV/AIDS reports, and distribution tables that we published for specific time frames (1985-2013) according to sex, age, age groups, and possible transmission routes were used and the groups were compared.

**Results:**

Although there were 35.3 (32.2-38.8) million people who were HIV(+) in the world as of 2013, only 9.7 million received AVTs. In Turkey, the total number of people with HIV/AIDS reported between 1985-2013 was 7050. There was a dramatic upward trend, with a peak in 2012 (n=1068). Sexually transmitted infection was the most common, and 4 drug use and blood transfusions showed a proportional increase. A total of 77 infections passed from mother to baby; seven cases have been reported in the last two years.

**Conclusion:**

Turkey is obliged to create an effective surveillance system for the prevention of HIV. The WHO proposed a new treatment protocol (option B+) in 2013 to prevent HIV mother-child transmission.

## INTRODUCTION

On the eve of the year 2013, 35.3 million (32.2-38.8 m) people were living with HIV throughout the world. The rate is likely to increase significantly compared with previous years because every year more people receive life-saving anti-viral therapy (AVT). Looking at 2001, across the globe it was seen that new cases of HIV, which amounted to 3.4 million (3.1-3.7 m), had decreased by 33% (2.3 million new cases of HIV)^(1)^. When death due to AIDS is considered, the rate has decreased from 2.3 million (2.1-2.6 m) in 2005 to 1.6 million (1.4-1.9 m) as of early 2013. The main cause of these results is that although incidence and mortality have decreased in Africa, they continue to increase in Western Europe, Central Asia, and the Mediterranean. Rates in other regions have remained the same^(2,3)^.

According to a report published in 2013 by the Republic of Turkey Ministry of Health, a total of 7050 people were reported to be HIV(+) from 1985 until November 2013. Males constitute about 73% of all cases. People aged between 40-49 years are those who are most affected by the infection. About 17% of those infected with HIV(+) in Turkey are foreign nationals. Sexual relations is the most common type of transmission in Turkey. HIV treatment is covered by social security in Turkey. Patients with HIV/AIDS who are not covered by health insurance are provided a green card, if their social situations are appropriate^(4)^.

Mother-to-child transmission: HIV can be transmitted to a child during pregnancy, childbirth, and breastfeeding. The probability is 20 to 30%. However, this ratio can be reduced to 1 or 2% if the HIV(+) mother receives treatment during pregnancy, birth is given at 39th week by cesarean section, and the baby receives treatment after birth and is under the care of physicians. Prevention of mother-to-child transmission of HIV is a very important field that is constantly evolving^(5)^. The latest treatment guidelines published by the World Health Organization (WHO) in 2013 emphasized how important it was for all pregnant women infected with HIV to receive life-prolonging AVT. The latest recommendations in 2013 stated that HIV-infected pregnant women should start to receive AVT (option B+) as soon as diagnosed, rather than following the 2010 guidelines in which the type of antiretroviral (ARV) treatment was determined and initiated based on eligibility criteria (option A and option B) of CD4 counts and disease severity^(1)^.

This study was intended to examine the epidemographic characterstics of HIV/AIDS and to report the epidemic data in terms of these between 1985 and 2013 in Turkey. Two years have passed since we published an article on the status of HIV in Turkey. In this study, we have focused on different aspects of the HIV/AIDS epidemic in Turkey and major problems that still exist. Furthermore, we present the management and treatment of pregnancy and HIV/AIDS in light of the current literature.

## MATERIALS AND METHODS

The Turkish Ministry of Health reports data that breakdown HIV-AIDS by age and sex, and possible transmission routes at 2-year intervals. The cases in this report are based on total data in the feedback system. In this study, data in these reports were analysed and grouped in five time periods. The figures from December 1996 have been subtracted from those of December 31^st^ 2001 in order to calculate 1997-2001 data. The same method was also for the other groups. Data after 2001 were grouped into 5-year periods. For the last group, data were used for two-year time frames between December 31^st^, 2011, and December 1^st^, 2013. The results for December 2013 had not yet been reported when this study was conducted. Age distribution, sex distribution, number of cases, and distribution of possible transmission routes have been calculated separately for all time periods.

## RESULTS

**Figure 1:**
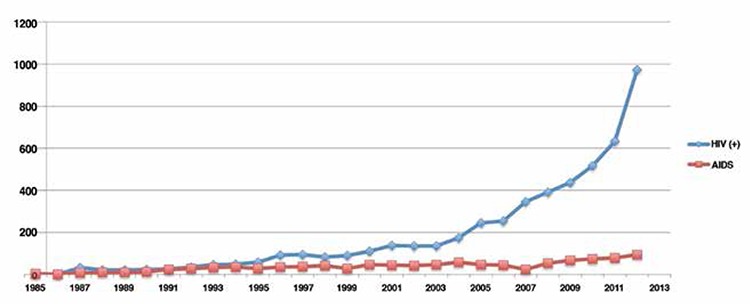
The distribution of HIV/AIDS cases reported in Turkey between 1985 and 2013, *(Red line) During reporting, cases which were clinically on stage of AIDS, **(Blue line) During reporting, HIV infection cases which were not clinically diagnosed as AIDS

When cases were examined without taking into account the December 2013 cases, which had not yet been reported by the Ministry of Health, the highest number of new cases per year was reported as 1068 for 2012. When we examined the reported HIV/AIDS cases according to the selected time periods, an upward trend was seen over the last ten years. The distribution of HIV/AIDS cases reported in Turkey between 1985 and 2013 is shown in Figure 1. The most common route of transmission was found to be heterosexual intercourse (Table 1). When distribution was examined between 1985-1996, heterosexual relationships were identified as the most common way of transmission (n=440, 57.7%), followed by intravenous^(4)^ drug use and blood transfusions in second place with 16.1%, homosexual sexual intercourse in third (14.8%), and mother-to-child transmission in fourth place (Table 1).

Between 2011-2013, the number of cases with an unknown route of transmission found to be 976; over time, especially after 2007, there has been an increase in inaccurately reported data. When incomplete data were subtracted from the total number of cases, infection through heterosexual relationship was found to be the most frequent transmission route between 2011-2013 (n=384, 21%). Using the same method, homosexual-bisexual relationships were found to be the second most frequent source of infection (n=220, 12%), followed by intravenous drug abuse (n=155, 8.4%), and nosocomial infection (n=85, 4.6%) (Table 1).

Considering the age groups, the rate of infection in patients aged 40 years and more had increased over time (Table 2). However, the disease was most prevelant in those aged 20-29 years, followed by patients aged 30-39 years. Mother-to-child transmission was found to have increased proportionally over time. Referring to the data of the last 2-year period, the ratio was 0.37% (n=7) for December 2012-2013. The are no data available on how many children who were infected via the mother-to-baby route received AVT.

## DISCUSSION

According to the decisions of the UN General Assembly in 2011, beginning from 2012, all member states should evaluate and improve their HIV surveillance systems, an advanced system and national data will be provided after being collected by these systems^(6)^. But still today, the Ministry of Health does not have a reliable and efficient data collection system for annual cases of HIV/AIDS. Accordingly, it is possible to criticize current reports published by the Ministry of Health in many ways. The most noticeable element is the large number of unknowns for each type of data. For example, looking at the distribution of HIV/AIDS cases in Turkey by period of reports in Table 2, the total number of cases in the age group of which is unknown is 508, and this corresponds to 7.2% (508/7050) of the total number of cases between 1985 and 2013. This constitutes a large bias when analyzing the data. Therefore, the reliability of the data is reduced. Another example for this is study time period; when transmission through intravenous drug addiction was analyzed in the study, it was found that it tended to decrease over time until 2011 as 3.7%, 2.1%, and 1.2%, respectively, whereas it was 11.5% between 1985 and 1996. However, it increased again to 8.4% between 2012-2013 (Table 1). Several studies in Turkey showed that the actual HIV/AIDS rates in intravenous drug users in Turkey may be even higher than the rates in the Ministry of Health’s annual reports for the last two years^(7,8)^.

Another important issue that arises from shortcomings in collection of HIV/AIDS-related data in Turkey is that the WHO has to report average values, the calculation method of which is not known. The organization cannot obtain information on many data such as the number of people treated, the number of people who underwent diagnostic tests, and the number of people who received education and information on his/her disease. Therefore, we believe that a discussion of these and other data in our study would bring some benefit, but we also believe that it is important to highlight the situation in order to show the extent of the shortcoming of reliable data collection. Thus, it is important for the results discussed in our study to be considered in light of this fact.

Although Turkey is among countries where HIV/AIDS prevalence is low, the incidence of infection in the country is tending to increase. In 2012, the number of new cases was the highest (n=1068) (Figure 1). The 2010 Turkey Development Goals declared targets of slowing the HIV epidemic between 2011-2014 and reducing the number of new annual cases to below 400^(9)^. According to the annual HIV/AIDS report announced by the Ministry of Health in December 2013, the number of new cases in 2013 was 835^(1)^. Although the goal has not been fully achieved, the decrease in the number of new cases, which was 1068 in 2012, was significant.

Transmission by blood transfusion significantly decreased over time The highest was 7.1% between 1985-1996, no such infection has been reported for the period 2012-2013. After the Law on blood products was published in 1983, unreliable practice continued until the 1990s^(10)^. Regulations covering checks of risky conduct of blood donors, and regular training of employees in blood transfusion centres began in 1996^(11)^. The first ever safe practices and standards were published much later in 2007^(12)^. The regulations explain the significant decrease in transmission by blood transfusion.

According to the UNAIDS 2013 Global Report, it was intended that 90% of HIV-infected pregnant women be given effective ARV treatment by 2015. This rate was 62% as of the year 2012. In 2012, the number of newly-infected children with HIV decreased by 35% compared with 2009. However, much more effort is foreseen to be necessary if the 2015 target for pregnant women and their children with HIV to access care and treatment is to be achieved^(1)^ (Table 3).

When the time periods in our study were examined proportionately, heterosexual and homosexual/bisexual infection through sexual intercourse were most common way of transmission. This increase was greater in heterosexual relationships. The rate was 57.7% (254/440) between 1985-1996, and increased to 81.3% (1410/1734) between 2007-2011, and decreased to 45.1% (384/851) in the last two years. Therefore, measures taken for safe sex will be the most important steps in reducing the transmission of HIV infection. Unfortunately, there are as yet no clear and effective steps in this regard in Turkey. There is no sex education for young people in schools. On the contrary, access to publications and web sites with sexual content has recently been prohibited. No source and internet access have been provided for science-based sex education and learning either. As we have noted in this study, sexually-transmitted diseases will continue to increase quickly unless social awareness regarding safe sex and sexually transmitted diseases is created, and campaigns and all other community-based information activities are developed.

This study has revealed that there was no effective activity for the purpose of controlling transmission from HIV-infected mothers to their children (Table 1). WHO stated in their 2011 progress report that there was no systemic activity in this regard in Turkey^(2,5)^.

HIV may be transmitted from the mother to child in the intrauterine period, during delivery, and after birth during breastfeeding^(13,14,15,16)^. Breast milk was considered to have played an important part in one-third or half of perinatal infections, especially in poorer regions such as Africa^(17)^. Infection is more likely with the milk given in colostrum and in the first 14 days because it has more viruses^(18)^. This review has revealed that data in Turkey in this respect are not reliable. The lack of a good data collection system makes the effective measures impossible to be taken.

Although not fully eliminated, the probability of transmission of disease from mother to child as a result of prophylaxis applied to HIV(+) pregnant women has been significantly reduced, and this encouraged many patients with HIV(+) to have a baby^(14)^. In countries where AVT is widely used, approximately one-third of people who know that they are HIV-infected are willing to have children and the number of HIV(+) women among them is greater than that of HIV(+) men^(19)^. When we conducted the review, we could find no other studies on this issue in Turkey. Any information on risk of infection and ways to protect should be transferred to patients with HIV(+) and their spouses who wish to have a child in order to be able to make sound decisions. If couples still want a child in view of this information, in cases where father is HIV(-), intravaginal or intrauterine insemination conception in the preovulatory phase is the most suitable^(20)^. If the father is HIV(+), conception is very difficult without risking infecting the mother. HIV has been reported to cause infection with insemination of donor semen^(21)^. Although reduced to a great degree by methods such as sperm washing and intracytoplasmic sperm injection, the rate of transmission from HIV(+) men to HIV(-) women and vertical transmission to children has not been fully eliminated. Moreover, these are costly processes that can only be carried out for research purposes in a special laboratory^(21)^.

It is mentioned in the literature that chorionic villus sampling during the intrapartum period, and invasive procedures such as amniocentesis, cordocentesis and obstetric factors such as premature birth, placenta previa, and presence of chorioamnionitis may increase the risk of perinatal HIV transmission(22). As most perinatal transmission occurs during birth, birth-related factors significantly affect risk of HIV transmission from mother to child. Before the start of labor and rupture of membranes, birth by elective cesarean section reduces the transmission of HIV from mother to infant^(14)^.

Almost all antiretroviral drugs are in group B or C of the United States Food and Drug Administration pregnancy category^(23)^. However, treatment may be interrupted in the first 14 weeks to minimize the potential teratogenic effects^(23,24)^. It is essential that all medication be discontinued altogether and started again altogether in order to avoid the development of resistance to drugs. Zidovudine is used as one of the drugs in the triple combination because it is the only antiretroviral agent that has been shown to reduce perinatal infection independent of reducing viral load^(14)^. The treatment of opportunistic infections that may arise in HIV(+) pregnant women is the same as in non-pregnant women with HIV infection^(23)^. Pneumococcal, hepatitis B, and inactivated influenza vaccines used for prophylaxis of these infections may also be adminstered to pregnant women if necessary, but rubella, measles, mumps, and varicella vaccines need to be avoided during pregnancy.

The WHO published a program update in April 2012 on HIV treatment in pregnant women and HIV prevention in infants, and most recently, in June 2013 it published a guide for the treatment and prevention of HIV infection^(1)^. In summary, the 2013 guide recommended to start AVT in all pregnant women with HIV and breastfeeding mothers in periods where there was transmission risk from mother to child and to continue for life. “Option A” is not recommended anymore^(25)^ (Table 4).

Turkey is a member state of “Euro HIV.” As such, it is obliged to conduct epidemiologic surveillance in determining the HIV epidemic in Europe. This should be conducted through an IT network in order for coordinated surveillance in Europe to be undertaken and its members need to develop their systems^(26)^. Thus, as declared in the Millennium Development Goals, Turkey is obliged to create an effective surveillance system for the prevention of HIV^(9)^.

As a result, this review revealed that significant differences existed in terms of HIV/AIDS transmission routes in Turkey between 1985 and 2013. Transmission through sexual intercourse played a dominant role in the HIV epidemic in Turkey. Sexual health education is needed for the whole of society, especially among young adults, to prevent the spread of HIV/AIDS. Diagnostic and screening tests should be included in maternal and child health programs to prevent mother-to-child transmission of HIV/AIDS. Among measures to minimize perinatal transmission risk are the reduction the mother’s viral load with antiviral drugs, reduction of exposure to genital secretions by elective cesarean section, shorten labor time as much as possible, avoid exposure after membrane rupture, and to restrict invasive procedures. The Ministry of Health should continue to publish annual HIV/AIDS cases with an advanced surveillance system that allows much more comprehensive data entry to minimize unknown data.

## Figures and Tables

**Table 1:**
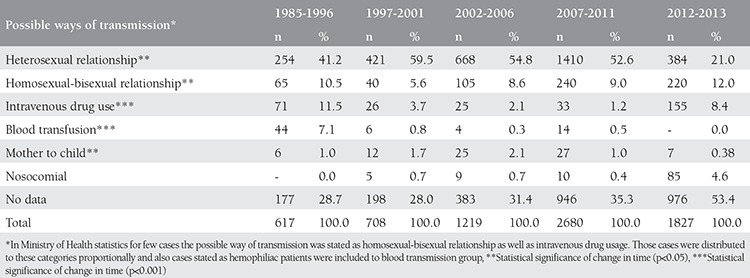
Distribution of possible transmission ways of HIV/AIDS cases in Turkey according to reporting period

**Table 2:**
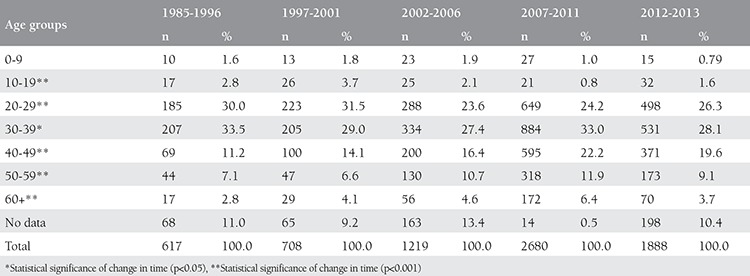
Age distribution of HIV/AIDS cases in Turkey according to reporting period

**Table 3:**
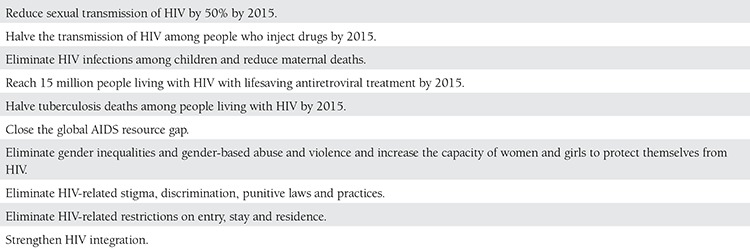
2011 UN Political Declaration on HIV/AIDS and elimination commitments for 2015

**Table 4:**
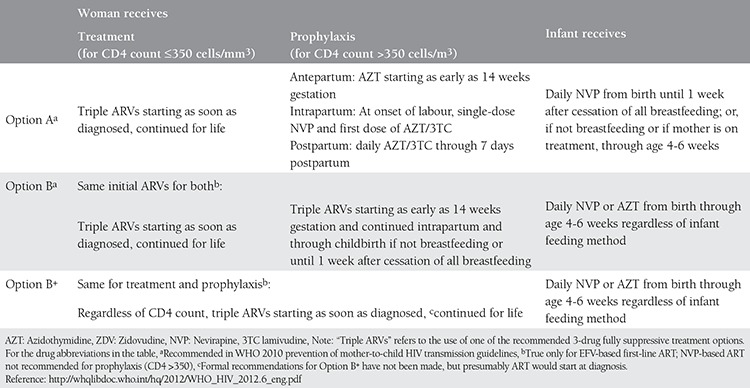
Three options for prevention of mother-to-child HIV transmission programmes

## References

[1] UNAIDS report on the global AIDS epidemic 2013, 4-14, 101.

[2] World Health Organization, UNAIDS, Unicef (2011). Global HIV/ AIDS response. Epidemic update and health sector progress towards Universal Access. Progress Report.

[3] no author (1997). Republic of Turkey Ministery of healt HIV/AIDS data tables 01 October 1985-01 December 2013(report) (Turkish). AIDS Prevention Bulltin.

[4] Republic of Turkey Ministery of healt HIV/AIDS 01 December 2013(report) (Turkish). 2013.

[5] Sherr L, Croome N, Parra Castaneda K, Bradshaw K (2014). A Systematic Review of Psychological Functioning of Children Exposed to HIV: Using Evidence to Plan for Tomorrow’s HIV Needs. AIDS Behav.

[6] no author (2011). UNAIDS global AIDS Response Progress Reporting 2012 Guidlines Construction of core indicators for surveillance the political declaration on. HIV/AIDS.

[7] Evren C, Tamar D, Ögel K, Çorapçıoğlu A, Çakmak D (2000). IV Heroin use and some related behavioral manner (Turkish). The Journal of Clinical Psychiatry.

[8] Saatçioğlu Ö, Evren EC, Çakmak D (2003). Evalution of inpatient cases with alcohol and drug use between years of 1998 and 2002. Journal of Dependence.

[9] no author (2010). TR Prime Ministry Undersecretariat of State Planning Organization, Office of the United Nations residect Coordinator. Milennium Development Goals Report Turley.

[10] np author (1983). Law and blood and blood products, no.2857 - (abolished) Accept date: 23.06. - issue date: 25.06.1983 (Turkish). TR Official Journal Number: 18088.

[11] Bayık M, Uluhan R, bayık M, Emektas G, Pelit NB (2009). The Process of Legistation, Regulations and Guidlines. National Blood Centers and Medicine of transfusion XII Advance Course Book.

[12] no author (2007). Law on blood and blood products, no:5624. Accept date: 11.04.2007 TR Official Journal Number: 26510 - issue date: 02.05.2007. (Turkish).

[13] European Collaborative Study (1992). Risk factors for mother-to-child transmission of HIV-1. Lancet.

[14] no author (2004). Public Health Service Task Force. Recomendations for the use of antiretroviral drugs in pregnant women infected with HIV-1 for maternal health and interventions to reduce perinatal HIV-1 transmisssion in the United States. Available at: http:/www.aidsinfo.nih.gov/guidelines/. Updated November 26 2003. Accessed June 3, 2004.

[15] Maury W, Potts BJ, Rabson AB (1989). HIV-1 infection of first trimestr and term human placental tissue; A possible mode of maternal-fetal transmission. J Infect Dis.

[16] Ehrnst A, Lindgren S, Dictor M, Johansson B, Sönnerborg A, Czajkowski J, et al (1991). HIV in pregnant women and their offspring; Evidence ffor late transmission. Lancet.

[17] Miotti PG, Taha TE, Kumwenda NI, Broadhead R, Mtimavalye LA, et al (1999). HIV transmission through breastfeeding: A study in Malawi. JAMA.

[18] Sperling RS, shapiro DE, Coobs RW, Todd JA, Herman SA, McSherry GD, et al (1996). Maternal viral load, zidovudine treatment, and the risk of transmission of human immunodeficiency virus type 1 from mother to infant. Pediatric AIDS Clinical Trials Group Protocol 076 Study Group. N Engl J Med.

[19] Chen Jl, Philips KA, Kanouse DE, Collins RL, Miu A (2001). Fertility desires and intentions of HIV-positive men and women. Fam Plann Perspect.

[20] Centers for Disease Control (CDC) (1990). HIV-1 infection and artificial insemination with processed semen. MMWR Morb Mortal Wkly Rep.

[21] Al-Khan A, Colon J, Palta V, Bardeguez A (2003). Assisted reproductive technology for men and women infected with human immunodeficiency virus type1. Clin Infect Dis.

[22] Zorrilla CD (1997). Obstetric factors and mother-to-infant transmission of HIV-1. Infect Dis Clin North Am.

[23] Guidelines for the use of Antiretroviral Agents in HIV-1 Infected Adults and Adollescents. April 7, 2005.

[24] no author (2004). HIP HIV/Pregnancy Clinical Practice Guidelines. Perinatal HIV Guideline Working Group, November 26,2003.Approved by Preventive Health Subcomittee. June.

[25] WHO, HIV/AIDS, Programmatic update use of antiretroviral drugs for treating pregnant women and preventing HIV infection in infants. April 2012, 1-8.

[26] Republic of Turkey Ministry of Health Turkey Strategic Plan for Improving the Communicable Diseases Surveillance and Control System 2009 - 2013 (Turkish).

